# Estimating mortality using data from civil registration: a cross-sectional study in India

**DOI:** 10.2471/BLT.15.153585

**Published:** 2015-11-02

**Authors:** Mamta Gupta, Chalapati Rao, PVM Lakshmi, Shankar Prinja, Rajesh Kumar

**Affiliations:** a School of Public Health, Post Graduate Institute of Medical Education & Research, Chandigarh 160012, India.; b Research School of Population Health, Australian National University, Canberra, Australia.

## Abstract

**Objective:**

To analyse the design and operational status of India’s civil registration and vital statistics system and facilitate the system’s development into an accurate and reliable source of mortality data.

**Methods:**

We assessed the national civil registration and vital statistics system’s legal framework, administrative structure and design through document review. We did a cross-sectional study for the year 2013 at national level and in Punjab state to assess the quality of the system’s mortality data through analyses of life tables and investigation of the completeness of death registration and the proportion of deaths assigned ill-defined causes. We interviewed registrars, medical officers and coders in Punjab state to assess their knowledge and practice.

**Findings:**

Although we found the legal framework and system design to be appropriate, data collection was based on complex intersectoral collaborations at state and local level and the collected data were found to be of poor quality. The registration data were inadequate for a robust estimate of mortality at national level. A medically certified cause of death was only recorded for 965 992 (16.8%) of the 5 735 082 deaths registered.

**Conclusion:**

The data recorded by India’s civil registration and vital statistics system in 2011 were incomplete. If improved, the system could be used to reliably estimate mortality. We recommend improving political support and intersectoral coordination, capacity building, computerization and state-level initiatives to ensure that every death is registered and that reliable causes of death are recorded – at least within an adequate sample of registration units within each state.

## Résumé

**Objectif:**

Analyser la conception et la situation opérationnelle du système indien d'enregistrement et de statistiques de l'état civil et favoriser l'évolution du système en une source précise et fiable de données sur la mortalité.

**Méthodes:**

Nous avons évalué le cadre juridique, la structure administrative et la conception du système national d'enregistrement et de statistiques de l'état civil à travers une étude de documents. Nous avons réalisé une étude transversale pour l'année 2013, à l'échelle nationale et dans l'État du Pendjab, afin d'évaluer la qualité des données du système sur la mortalité; à cet effet, nous avons analysé les tables de mortalité et examiné le caractère complet des enregistrements de décès ainsi que la proportion de décès inscrits comme étant dus à des maladies. Nous avons interrogé des officiers de l'état civil, des médecins et des codeurs de l'État du Pendjab afin d'évaluer leurs connaissances et leur pratique.

**Résultats:**

Bien que le cadre juridique et la conception du système aient été appropriés, le recueil de données reposait sur des collaborations intersectorielles complexes, au niveau local et de l’État, et les données recueillies étaient de mauvaise qualité. Les données enregistrées ne permettaient pas d'estimer correctement la mortalité au niveau national. Une cause de décès certifiée médicalement n'était notée que pour 965 992 (16,8%) des 5 735 082 décès enregistrés.

**Conclusion:**

Les données enregistrées en 2011 par le système indien d'enregistrement et de statistiques de l'état civil étaient incomplètes. Une fois amélioré, ce système pourrait permettre de faire une estimation fiable de la mortalité. Nous recommandons de renforcer le soutien politique ainsi que la coordination intersectorielle, les compétences, l'informatisation et les initiatives menées à l'échelle de l'État afin de s'assurer que chaque décès soit enregistré et que des causes de décès fiables soient notées – au moins dans une partie des unités d'état civil de chaque État.

## Resumen

**Objetivo:**

Analizar el diseño y estado de funcionamiento del sistema de registro civil y de estadísticas vitales de India y facilitar el desarrollo del sistema hacia una fuente precisa y fiable de datos de mortalidad.

**Métodos:**

Se evaluó el marco legal, la estructura administrativa y el diseño del sistema nacional de registro civil y de estadísticas vitales a través de la revisión de documentos. Se llevó a cabo un estudio transversal del año 2013 a nivel nacional y en el estado de Punjab para evaluar la calidad de los datos de mortalidad del sistema a través del análisis de tablas de mortalidad y se investigó la exhaustividad del registro de defunciones y la proporción de defunciones atribuidas a causas no definidas. Se entrevistó a agentes de registro, oficiales médicos y codificadores del estado de Punjab para evaluar sus conocimientos y experiencia.

**Resultados:**

Aunque se observó que el marco legal y el diseño del sistema eran apropiados, la recopilación de datos estaba basada en colaboraciones intersectoriales complejas a nivel estatal y local y se descubrió que los datos recogidos eran de mala calidad. Los datos de registro eran inadecuados para una buena estimación de la mortalidad a nivel nacional. Solo se documentó una causa de muerte médicamente certificada para 965.992 (16,8%) de las 5.735.082 defunciones registradas.

**Conclusión:**

Los datos registrados por el sistema de registro civil y de estadísticas vitales de India en 2011 fueron incompletos. Si se mejorase, el sistema podría utilizarse para estimar la mortalidad de forma fiable. Se recomienda mejorar el apoyo político y la coordinación intersectorial, el aumento de capacidad, la informatización y las iniciativas a nivel estatal para asegurar que se registran todas las defunciones y que se documentan causas de muerte fiables, al menos dentro de unas unidades de muestra de registro adecuadas para cada estado.

## ملخص

**الغرض:**

تحليل خطة نظام التسجيل المدني والإحصاءات الحيوية والوضعية التنفيذية له بالهند وتيسير تطوير هذا النظام ليصبح مصدرًا دقيقًا وموثوقًا به فيما يتعلق ببيانات الوفيات.

**الطريقة:**

أجرينا تقييمًا لنظام التسجيل المدني والإحصاءات الحيوية على المستوى المحلي فيما يتعلق بالإطار القانوني والهيكل الإداري والخطة الخاصة به، وذلك من خلال مراجعة الوثائق. وأجرينا دراسة متعددة القطاعات لعام 2013 على المستوى المحلي وفي ولاية البنجاب لتقييم جودة بيانات الوفيات بالنظام من خلال تحليلات جداول الوفيات واستقصاء اكتمال تسجيل حالات الوفاة ونسبة حالات الوفاة المسندة إلى أسباب تفتقر إلى دقة التحديد. وأجرينا مقابلات مع موظفي السجل المدني، ومسؤولي الخدمات الطبية، ومسؤولي الترميز الإحصائي في ولاية البنجاب لتقييم ما لديهم من معلومات ولتقييم ممارساتهم.

**النتائج:**

على الرغم من أننا رأينا أن الإطار القانوني وخطة النظام ملائمان، اعتمد جمع البيانات على أنشطة تعاونية معقدة فيما بين القطاعات على مستوى الولاية وعلى المستوى المحلي، كما أننا توصلنا إلى أن البيانات التي تم جمعها شابها تدني مستوى الجودة. وكانت البيانات المتوفرة بنظام التسجيل غير ملائمة لإجراء تقييم متقن لمعدل الوفيات على المستوى المحلي. وتم فقط تسجيل سبب الوفاة الصادر به شهادة طبية لعدد من حالات الوفاة يبلغ 965,992 (16.8%) من بين 5,735,082 من حالات الوفاة التي تم تسجيلها.

**الاستنتاج:**

لقد شاب النقصان البيانات المسجلَّة من جانب نظام التسجيل المدني والإحصاءات الحيوية بالهند لعام 2011. وإذا تم تحسينها، يمكن استخدام هذا النظام لتقدير معدل الوفيات على نحو جدير بالثقة. ونوصي بتحسين الدعم السياسي والتنسيق فيما بين القطاعات، وبناء القدرات، واستخدام أجهزة الكمبيوتر، والمبادرات على مستوى الولاية لضمان تسجيل كل حالة وفاة وتدوين أسباب الوفاة الموثوق بها - في نطاق عينة وافية على الأقل من وحدات التسجيل داخل كل ولاية.

## 摘要

**目的:**

旨在分析印度户籍和生命统计体系的设计和运行状态，促使该体系成为精确可靠的死亡数据来源。

**方法:**

通过文档审核，我们评估了该国户籍和生命统计体系的法律框架、管理结构和设计。根据 2013 年的国家数据，我们在旁遮普邦展开了一项横断面调查，通过分析生命表和调查死亡登记的完整性以及被认定为因不明原因而死亡的比例，评估该体系死亡数据的精确性。

我们访问了旁遮普邦户籍员、卫生官员和编码员，以评估其知识和实践能力。

**结果:**

尽管我们发现法律框架和体系设计合理，但是由于数据收集仍基于邦和地方复杂的跨部门合作，我们发现所收集的数据仍不准确。登记数据无法用于精确估计全国死亡率。根据记录，登记的 5 735 082 例死亡中，死因经医学证明的案例仅为 965 992 例 (16.8%)。**结论 **印度户籍和生命统计体系 2011 年记录的数据不完整。如果经过改进，可使用该体系对死亡率进行可靠评估。我们建议改进政策支持和跨部门协作，能力构建、计算机化和邦级举措，确保登记每一例死亡并记下可靠的死因——至少在各邦登记部门的精确样例中照此登记。

## Резюме

**Цель:**

Проанализировать строение и рабочее состояние системы регистрации актов гражданского состояния и статистики естественного движения населения в Индии и содействовать превращению системы в точный и достоверный источник данных о смертности.

**Методы:**

Путем изучения документации нами была проведена оценка юридической базы, на основании которой функционируют система регистрации актов гражданского состояния и статистики естественного движения населения, а также их административная структура и схема. В течение 2013 года было проведено одномоментное поперечное исследование на национальном уровне и на уровне штата Пенджаб для оценки качества данных о смертности, предоставляемых системой; оценка проводилась путем анализа таблиц смертности по поколениям и изучения полноты записей при регистрации смерти, а также путем определения доли смертей, для которых причины были определены неточно. Были опрошены сотрудники ЗАГСов, медицинские сотрудники и кодировщики, работающие в штате Пенджаб, для оценки их знания и практических методов работы.

**Результаты:**

Хотя юридическая база и организационная схема системы были оценены как приемлемые, сам сбор данных на уровне штата и на местном уровне опирался на сложную систему межсекторальных взаимодействий и собранные данные оказались низкого качества. Данные регистрации не удовлетворяли требованиям твердой оценки смертности на национальном уровне. Из 5 735 082 зарегистрированных смертей медицинская справка о причине смерти была выдана только в 965 992 случаях (16,8%).

**Вывод:**

Данные, зарегистрированные в системе записи актов гражданского состояния и статистики естественного движения населения Индии в течение 2011 года, были неполными. При условии внесения улучшений система может послужить для достоверной оценки уровня смертности. Рекомендуется улучшить политическую поддержку на уровне стратегического планирования и межсекторальной координации, развивать мощности, внедрять компьютеризацию и проявлять инициативы на уровне штатов, чтобы гарантировать регистрацию каждой смерти с указанием достоверно установленной ее причины хотя бы в пределах соответствующей выборки по регистрационным отделениям в каждом штате.

## Introduction

Vital statistics are essential for tracking population dynamics, assessing health risks and evaluating health programmes.^1^ In India, a national civil registration and vital statistics system – hereafter called the vital statistics system – is still under development. Estimates of mortality are based on alternate data sources – e.g. censuses, the sample registration system and specific projects.^2^ Fragmentary data from such sources have been used recently to derive national estimates of mortality by age, sex and cause.^3^^–^^5^ Inconsistency between estimates has hampered the evaluation of burdens posed by malaria, human immunodeficiency virus and road traffic collisions.^6^^–^^8^

Before the introduction of the Registration of Births and Deaths Act in 1969, registration was non-uniform across states in India. The current system is supported by a national agency – the Office of the Registrar General of India– and registrars at state and local level.^9^ The accuracy of the system, particularly in relation to cause of death, is limited.^10^

In 1964–1965, the Office of the Registrar General introduced the sample registration system – as a separate entity from the vital statistics system – to measure fertility and mortality rates at national and state level in both urban and rural areas. In 2013, the sample registration system covered 7597 primary registration units with a combined population of 7.52 million.^11^ In urban areas, the Registration of Births and Deaths Act of 1969 requires attending physicians to certify causes of deaths under the medical certification of cause of death scheme^10^ – hereafter called the certification scheme. Although this scheme’s coverage has gradually expanded over the last four decades, it remains patchy.^12^ Between 1962 and 1999, causes of rural deaths were investigated in several surveys implemented by the Office of the Registrar General in collaboration with state health ministries.^13^ In selected primary health centres, paramedical staff used disease-specific algorithms and a structured questionnaire on symptoms and signs to assign causes of death. The job of ascertaining causes of deaths in rural areas was transferred to the sample registration system in 1999.^13^ Since then, the sample registration system has piloted verbal autopsy procedures and reported national summaries of causes of death as part of the Million Deaths Study.^14^^,^^15^ Each year, this system captures barely 0.5% of the estimated deaths in India. The current sample is sufficiently powered to measure infant mortality reliably but is inadequate to provide accurate measures of child, maternal or adult mortality or life expectancy at state or district level.

Given its nationwide coverage, a strengthened vital statistics system could meet the growing needs for detailed, timely and reliable data on mortality. Therefore, we investigated the system’s design and functional status to facilitate the system’s development into an accurate and reliable source of mortality data.

## Methods

We used several sources to assess the design of the vital statistics system and to analysis of broad characteristics of the system’s performance at national and subnational level (Box 1).^16^^–^^25^ The quality of coverage was assessed in terms of the completeness of death registration and the quality of the recorded causes of death.

Box 1 **Sources of data used in the assessment of the civil registration and vital statistics system, India**

- The Registration of Births and Deaths Act, 1969.^16^
- Model registration rules for Punjab state, 1999.^17^
- Registrar General of India’s report on vital statistics, 2011.^18^
- Population by age and sex for each state from 2011 census.^19^
- Report on life tables from the sample registration system.^20^
- Report on medical certification of cause of death.^12^
- Registration data for Punjab state and districts, 2012.^21^^,^^22^
- Vital statistics of India based on the civil registration system 2008, 2009 and 2010.^23^^–^^25^

The completeness of death registration for individuals aged at least six years at time of death was estimated by two methods: (i) using the number of deaths reported to the vital statistics system^18^ and the Brass growth balance indirect demographic technique;^26^ and (ii) applying the sample registration system’s state-level mortality rates for 2011^11^ to the state populations recorded in the 2011 census, to estimate the total mortality at national scale (the denominator).^19^ The number of deaths registered in the civil registration system is the numerator. Dividing the numerator by the denominator gives the percentage completeness of death registration. In this way, we analysed the trend in the completeness of death registration for the civil registration system from 1999 to 2011.^23^^–^^25^ State specific estimates were computed from life tables^27^ based on the 2011 census population and the 2011 vital statistics system reported deaths, without any adjustments for incomplete death registration. The quality of the medically certified causes of death was considered to be good if the proportion of underlying causes assigned codes representing only symptoms, signs and ill-defined conditions was less than 10%.^28^

The vital statistics system’s functional aspects were assessed in three districts of Punjab state, where the health sector is responsible for the system’s implementation. For the year 2012, the districts of Fatehgarh Sahib, Bathinda and Amritsar were selected to represent three levels of completeness of death registration: less than 80%, 80–90% and more than 90%, respectively.^21^ Information on the infrastructure was collected from a stratified random sample of 21 primary registration units that, together, covered primary, secondary and tertiary health facilities in both urban and rural settings. In each registration unit, a preliminary questionnaire was used to record information on registration operations. The target sample size for the evaluation of the completeness and accuracy of the recorded data – i.e. 300 records each for births, deaths and causes of death – was based on the expectation that, within a 10% margin of error at a 95% level of confidence, 70% of the records would be accurate and complete. A sample of 15 birth and death reports for 2013 was randomly selected from each of the 21 study registration units before the corresponding entries in the other relevant registers and reports were scrutinized. In addition, 80 certification scheme forms from each district hospital in the study area and 100 from the teaching hospital in the study area were evaluated.

Documents were evaluated for the completeness, accuracy and consistency of the recorded information across individual reports, registers and monthly returns. A record of a live birth or stillbirth was only considered complete if it noted the maternal age, date and place of birth, sex, parity, birth weight and address of the mother. A death record was only considered complete if it noted the date, place and cause of death and the type of medical attention.

We did semi-structured interviews with seven registrars, four medical officers and four medical staff who did coding, to evaluate their knowledge of, attitudes towards, and practices within the vital statistics system.

## Results

### Administrative aspects

The Registration of Births and Deaths Act of 1969 mandates compulsory registration, provides definitions of vital events and key terms and recommends registration formats and processes for statistical compilation. At state level, model registration rules elaborate on the operational aspects. In general, the head of the affected household and/or the village headman is responsible for notification of births and deaths in the community whereas the attendant physicians are responsible for notification of such events when they occur in health facilities. However, the Act mentions several additional notifiers – e.g. community health staff and local police – who may be among the first people to observe a death. The dead person’s usual place of residence – as well as the place of birth or death – should be noted. There are fines for non-compliance.^16^^,^^17^

While the legal framework is comprehensive, it permits the registration of death after cremation or burial, fails to call for periodic standardized inspections of the primary registration units, and fails to provide adequate detail on data standards – particularly in terms of the registration of causes of death.^16^^,^^17^

**Fig. 1:**
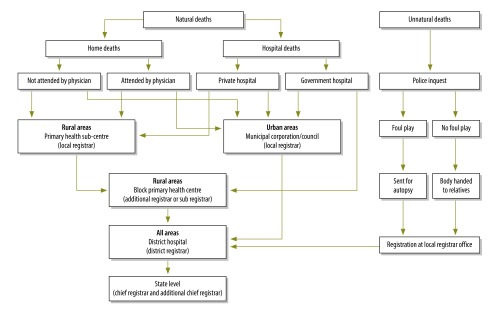
Organizational flowchart of the civil registration and vital statistics system to report deaths in Punjab, India, 2013

India’s vital statistics system follows a model of decentralization with multiple-level administration.^29^ Although the system is led by the Office of the Registrar General, each state has an independent structure led by a nominated state registrar from India’s administrative or health sectors or the Statistics Office. Within each state, registration is delegated to local government units from different sectors (Table 1). Only Punjab (Fig. 1) and eight other states have a single sector that holds responsibility for the vital statistics system at both state and local level. Funding for some of the vital statistics system’s components – e.g. training, stationery, computers and photocopiers – is provided by the State Directorate of Census Operations. The latter is a subordinate office of the Office of the Registrar General that does not have any operational role in the vital statistics system. The complexity of the system’s organizational structure underscores the need for close intersectoral collaboration.

**Table 1:** Design and functional status of death registration systems, India, 2011

Area	Population (millions)	Sector implementing system		No. of deaths registered by CRVSS	Estimates for males/females			CRVSS reporting coverage in rural/urban areas, %^b^	Estimates of CRVSS completeness, % of deaths			Medically certified deaths	
		State level	Sub-district level		Adult mortality,^a^ deaths per 1000 population	Life expectancy at birth, years			Based on CRVSS data^c^	Based on SRS data^d^		Total no.	No. of ill-defined causes (%)
						Based on CRVSS data	Based on SRS data						
**Major state^e^**													
Andhra Pradesh	84.6	Health	Admin	420 646	120/90	76/84	64/68	72/100	46	66		66 442	6 179 (9.3)
Arunachal Pradesh	1.4	Statistics	Admin	1 560	NA	NA	NA	NA	NA	22		507	12 (2.4)
Assam	31.2	Health	Health	111 054	NA	NA	61/63	91/100	NA	45		16 160	0 (0.0)
Bihar	104.1	Statistics	Admin^f^	155 176	NA	NA	65/66	NA	NA	24		7 429	334 (4.5)
Chhattisgarh	25.5	Statistics	Admin	114 842	NA	NA	NA	73/100	NA	60		9 550	974 (10.2)
Delhi	16.8	Statistics	Health	112 142	211/121	62/70	NA	100/100	40	100		63 611	17 302 (27.2)
Goa	1.5	Statistics	Admin	11 326	209/75	66/73	NA	100/100	50	95		11 321	1 574 (13.9)
Gujarat	60.4	Health	Admin	324 080	148/75	68/76	65/69	100/100	54	82		70 275	15 461 (22.0)
Haryana	25.4	Health	Health	153 530	NA	NA	67/69	100/100	NA	92		NA	NA
Himachal Pradesh	6.9	Health	Admin	42 524	134/72	69/76	68/72	100/100	59	93		5 014	266 (5.3)
Jammu and Kashmir	12.5	Health	Police	35 425	55/57	91/100	69/71	60/56	38	55		244	0 (0.0)
Jharkhand	33.0	Statistics	Admin^f^	116 615	NA	NA	NA	NA	NA	54		412	28 (6.8)
Karnataka	61.1	Statistics	Admin	384 745	171/88	67/75	65/70	95/97	57	91		123 221	1 972 (1.6)
Kerala	33.4	Admin	Admin	244 295	129/49	69/76	72/77	100/100	54	100		29 252	380 (1.3)
Madhya Pradesh	72.6	Statistics	Admin^f^	351 621	137/112	74 / 82	61/64	96/99	55	60		37 131	9 877 (26.6)
Maharashtra^g^	112.4	Health	Admin	633 206	262/147	70/76	68/72	92/95	53	88		215 618	24 365 (11.3)
Manipur	2.9	Health	Health^h^	4 253	NA	NA	NA	57/40	NA	42		2 215	128 (5.8)
Meghalaya	3.0	Health	Health	14 848	170/126	70/79	NA	100/91	42	72		1 681	178 (10.6)
Mizoram	1.1	Admin	Education	5 484	196/80	69/79	NA	87/84		100		2 537	104 (4.1)
Nagaland	2.0	Admin	Education	6 961	114/105	91/86	NA	93/94	37	93		162	0 (0.0)
Odisha	42.0	Health	Health	277 484	149/115	68/72	62/64	NA	57	80		33 975	2 582 (7.6)
Punjab	27.7	Health	Health	187 675	167/102	68/76	67/72	100/100	69	99		19 920	1 614 (8.1)
Rajasthan	68.5	Statistics	Education	360 560	134/92	69/75	65/68	96/100	80	79		42 417	1 824 (4.3)
Sikkim	0.6	Health	Health	3 094	162/104	70/77	NA	100/100	44	90		1 275	31 (2.4)
Tamil Nadu	72.1	Health	Admin	476 709	183/90	68/75	67/71	99/99	43	95		149 946	3 059 (20.4)
Tripura	3.7	Health	Admin	8 911	NA	NA	NA	100/100	NA	49		4 553	961 (21.1)
Uttar Pradesh	199.8	Health	Health	751 596	NA	NA	62/64	NA	NA	47		NA	NA
Uttarakhand	10.1	Health	Health	29 300	NA	NA	NA	75/78	NA	47		3 800	319 (8.4)
West Bengal	91.3	Health	Admin	371 079	NA	NA	67/71	88/75	NA	67		21 484	494 (2.3)
**All India**	1210.2	Admin	–	5 735 082	NA	NA	65/68	92/96	NA	67		965 992	118 817 (12.3)

Admin: administrative; CRVSS: civil registration and vital statistics system; NA: not available; SRS: sample registration system.

^a^ Probability of dying between ages 15–60 years per 1000 population.

^b^ Defined as the percentage of all rural/urban registration units throughout the state that submitted monthly and annual reports.^18^

^c^ Estimated using the Brass growth balance indirect demographic technique.^26^

^d^ Estimated by applying SRS state-level mortality rates to the state populations recorded in 2011 census.

^e ^This excludes the Union Territories of India.

^f^ With the health sector in a secondary role, supporting the administrative sector.

^g^ The CRVSS mortality data reported here for Maharashtra were collected in 2010.

^h^ With the administrative sector in a secondary role, supporting the health sector.

In 2001, the Office of the Registrar General issued separate instructions from the Registration of Births and Deaths Act and the sample registration system, on the compilation of vital statistics and called for detailed tabulations of deaths by age and sex from each state.^30^ Guidelines and formats for the standardized coding and compilation of data on causes of death have also been issued as part of the certification scheme.^31^

### Technical perspectives

Notifications for live births, stillbirths, deaths and causes of death conform to international standards.^32^ Medically certified causes of death are coded according to the International Classification of Diseases, tenth revision (ICD-10).^33^^,^^34^ In practice, there are several areas in which data completeness or quality could be improved. For 2–5% of registered deaths, for example, sex and/or age is not recorded.^12^ In India, the oldest age group considered in the summary statistics on deaths is older than 70 years whereas international standards require it to be older than 85 years.^33^ Although place of death is usually recorded, place of usual residence is often missing.^33^^,^^34^

The data from Punjab revealed that, although rural health centres generally appeared to be adequately staffed for registration, less than 50% of the statistical staff positions available within the state’s health sector were filled. Most (3/4) of the hospitals we investigated had no staff designated for the ICD-10 coding of causes of death. Most of the local registrars we interviewed had an inadequate understanding about the filling of forms, the registration of stillbirths and the processing of delayed registrations. Fifteen (71%) of the 21 study registration units had never received a visit by a district or state official who wished to assess the quality of their registration data. The field personnel we interviewed generally believed that the training programmes associated with the vital statistics system were too theoretical and lacked practical field-based exercises. Many of the nurses and pharmacists trained in coding causes of death had subsequently found themselves to be uninvolved in such coding.

### Data quality

Close to six million deaths – i.e. more than two-thirds of the 8 503 372 deaths estimated to occur annually^18^ – were registered in India’s vital statistics system during 2011. Table 1 presents summary mortality indicators for India’s major states. The operational functionality of the system is indicated by the high levels of reporting coverage across India. The detailed data needed for the construction of life tables were available for two-thirds of the states.

**Fig. 2:**
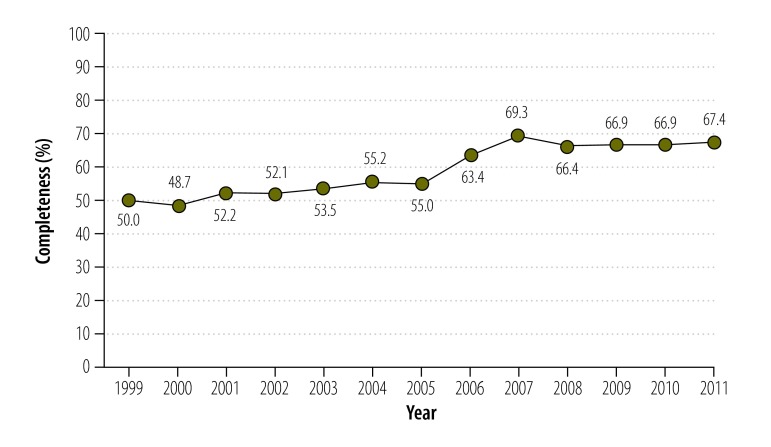
The completeness of death registration, India, 1999–2011

Notes: Completeness was calculated as the percentage of the estimated number of deaths occurring in each year. This number was estimated using data recorded by the sample registration system.

The mortality estimates indicated a gradual improvement in the completeness of death registration between 1999 and 2011 (Fig. 2) – but these were fairly crude as they took no account of the variations arising from sample distribution, sampling error or sex or age differentials. Estimates of life expectancy based on the vital statistics system were sometimes implausibly high – and often much higher than the estimates based on the sample registration system – probably because of the relative incompleteness of the data in the vital statistics system.

**Fig. 3:**
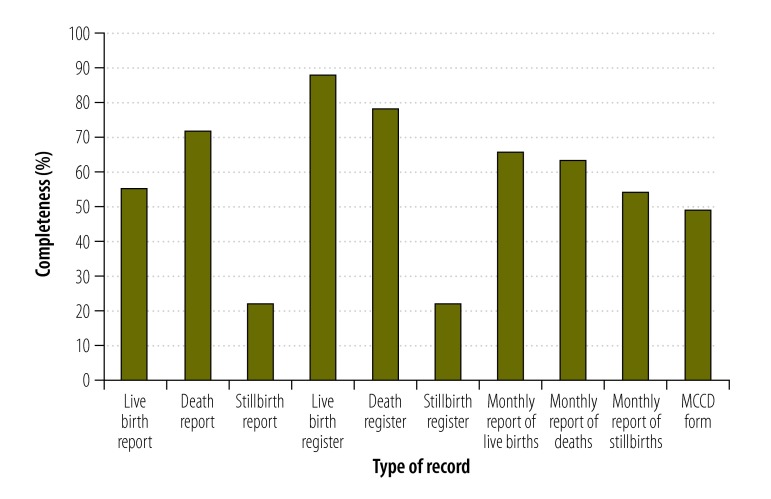
Completeness of the vital statistics records in three districts of Punjab, India, 2013

The results of the district-level analysis from Punjab showed that (Table 2) indicate disproportionately high numbers of registered deaths in Amritsar, Faridkot, Jalandhar and Ludhiana, probably because of the preferential utilization of tertiary care in these cities by people from the surrounding districts. The death-related data in the vital statistics system were sufficiently complete to allow estimates of the age- and sex-specific levels of mortality even at district level (Table 2). However, there were more registered deaths among men than women and there were problems with the quality of the registration documents. For example, the data for most stillbirths and almost half of the live births were recorded incompletely (Fig. 3). The recorded information for key variables was found accurate in 95% (285/300) of the birth register’s forms and in 83% (249/300) of the death register’s forms. For neonatal and infant deaths, it was rare to record age at death in terms of months, days and hours. On death reports, the columns for recording information on specific factors – e.g. pregnancy, smoking and alcohol use – were usually left empty. Even if such information was recorded on a death form, there was no space for it on the corresponding death register. Death registers only captured the legal variables required for the issuance of death certificates. Of the certification scheme forms that we evaluated, over half (163/300) recorded an ill-defined condition – e.g. heart failure or cardiopulmonary arrest – as the underlying cause of death.

**Table 2:** Mortality and life expectancy in the districts of Punjab, India, 2012

District	Population (millions)	No. of deaths registered by CRVSS	Estimates for males/females		CRVSS completeness, %
			Adult^a^ deaths per 1000 population	Life expectancy at birth, years^b^	
Amritsar	2.5	21 473	172/124	64/69	94
Bathinda	1.4	8768	149/90	71/81	65
Barnala	0.6	3862	161/96	70/76	66
Faridkot	0.6	5251	222/140	67/73	66
Fatehgarh Sahib	0.6	3378	140/71	72/77	81
Ferozepur	2.0	8346	108/72	74/80	49
Gurdaspur	2.3	14 529	121/77	70/76	81
Hoshiarpur	1.6	11 836	174/109	68/75	66
Jalandhar	2.2	19 356	199/172	63/69	71
Kapurthala	0.8	4897	129/89	74/84	57
Ludhiana	3.5	26 538	307/221	69/77	45
Mansa	0.8	4960	193/68	69/76	73
Moga	1.0	7028	111/90	70/74	88
Muktsar	0.9	5435	128/125	69/66	70
Patiala	1.9	13 380	74/54	68/72	96
Roopnagar	0.7	4597	150/63	69/75	69
Sahibzada Ajit Singh Nagar	1.0	5870	154/95	70/76	58
Sangrur	1.7	11 069	147/93	70/76	73
Shahid Bhagat Singh Nagar	0.6	5281	219/133	64/75	61
Tarn Taran	1.1	8028	210/128	66/73	82

CRVSS: civil registration and vital statistics system.

^a^ Probability of dying between ages 15–60 years per 1000 population.

^b^ Based on the CRVSS data.

^c^ Estimated from the CRVSS data, using the Brass growth balance indirect demographic technique.^26^

MCCD: medical certification of causes of death.

Notes: A record of a live birth or stillbirth was only considered complete if it noted the maternal age, date and place of birth, sex, parity, birth weight and address. A death record was only considered complete if it noted the date, place and cause of death and the type of medical attention.

A medically certified cause was recorded for just 965 992 (16.8%) of the 5 735 082 deaths registered in 2011 (Table 1). The most recent data available on causes of death – including the leading causes for 2011 (Table 3) – come from the certification scheme. In 2011, vague or ambiguous categories such as “ill-defined cause”, “septicaemia” and “other heart disease” accounted for nearly a quarter of all medically certified causes. In several states more than 10% of deaths were assigned to ill-defined causes (Table 1). The failure to assign an accurate cause of death reduces the value of all of the data on cause of death.^35^ Furthermore, since most of the deaths with medically certified causes occurred in health facilities, the data collected on causes of death may not reflect broader national trends.

**Table 3:** Leading causes of death recorded within the medical certification of causes of death scheme, India, 2011

Rank	Males (*n* = 587 375)				Females (*n* = 364 403)		
	Recorded cause	ICD-10 codes	% of medically certified deaths		Recorded cause	ICD-10 codes	% of medically certified deaths
1	Other heart disease	I26–I51	10.6		Other heart disease	I26–I51	11.1
2	Ischaemic heart disease	I20–I25	9.2		Ischaemic heart disease	I20–I25	8.7
3	Perinatal condition	P00–P96	7.1		Perinatal condition	P00–P96	7.7
4	Cerebrovascular disease	I60–I69	4.6		Cerebrovascular disease	I60–I69	4.5
5	Respiratory tuberculosis	A15–A16	4.1		Septicaemia	A40–A41	4.5
6	Septicaemia	A40–A41	3.9		Hypertensive disease	I10–I14	3.9
7	Diseases of the liver	K70–K76	3.8		Diabetes mellitus	E10–E14	3.8
8	Chronic lower respiratory disease	J40–J47	3.6		Chronic lower respiratory disease	J40–J47	2.9
9	Diabetes mellitus	E10–E14	3.4		Respiratory tuberculosis	A15–A16	2.8
10	Hypertensive diseases	I10–I14	3.3		Renal failure	N17–N19	2.3
11	Symptoms or ill-defined condition	R00–R99	12.4		Symptoms or ill-defined conditions	R00–R99	12.7

ICD-10: International Classification of Diseases, tenth revision.

An attempt was made to identify and evaluate the major sources of information on the causes of deaths occurring in Punjab (Table 4). None of the five identified sources was found to be adequate.

**Table 4:** Sources of cause of death statistics in Punjab, India, 2013

Characteristic	Data source				
	Civil registration and vital statistics system		National Health Mission’s report to Ministry of Health and Welfare	Punjab Health System Corporation’s health facility report	Sample registration system’s verbal autopsy reports
	Death reports	Medical certifications			
Completeness,% of deaths (*n* = 189 571)	99.0 (187 675)	10.3 (19 620)	35.6 (67 538)	5.0 (9 433)	1.8 (3 378)
Most recent data available, year	2011	2010	2012	2009	2001–2003
Source of cause of death	Lay individuals	Attending physicians	Lay individuals or paramedical staff	Medical record cover sheets	Physician review of verbal autopsies
% of deaths attributed to ill-defined causes (no. ill-defined causes/all causes)	NA^a^	7.4 (1 452/19620)	50.0 (33 769/67538)	8.0 (755/9433)	9.9 (338/3378)
Limitations of data source	Cause given in non-medical terms	Not representative of either urban or rural population. Lacking a defined source population	Confined to rural settings	Excludes deaths at home	District-level and block-level data not available

NA: not available.

^a^ Computerized data from Shahid Bhagat Singh Nagar district indicates that 60% of the deaths recorded in the district were assigned as ill-defined causes.

### Societal perspectives

The Indian Government has undertaken several initiatives to strengthen the vital statistics system. In some states, the certification scheme requires all health facilities to register in-facility deaths. The government-led computerization of all birth and death reports, which has been initiated in some districts of a few states, offers the possibility of linking data from different sources. National and state-level interdepartmental committees have been established to review the performance of the vital statistics system annually.

At community level, we did not come across any direct evidence of targeted campaigns to increase public awareness of the vital statistics system except for a few public notices. Although communities increasingly recognize the need for death certificates for adults – e.g. to effect property transfers and other legal or financial transactions – they appear to be less inclined to seek death certificates for infants or appreciate the importance of the accurate reporting of causes of death.

## Discussion

For several states, mortality data for 2011 from the vital statistic system were available in sufficient detail to enable life-table analyses – albeit with known biases. Despite considerable heterogeneity in the vital statistics system’s administrative structure and organization – both among and within states – the data collected by the system indicate that functional intersectoral collaboration exists but needs to be strengthened, perhaps through re-alignment. For example, there was a considerable increase in data capture after Haryana state transferred responsibilities for birth and death registration from the police to the health sector in 2005.^36^To enhance coordination, the Ministry of Health and Family Welfare – in collaboration with the Office of the Registrar General – convened an interdepartmental coordination committee. The members of this committee, who met several times in 2012,^37^ made several important recommendations that could strengthen the vital statistics system (Box 2). Any such strengthening activities need to emphasize the roles and responsibilities of private health facilities and personnel in the registration of births, deaths and causes of death.

We did not present any findings on deaths among children younger than six years because few deaths were registered in this age group. A protocol for the routine review of maternal and child deaths has recently been developed to identify the relevant gaps in health service delivery.^38^^,^^39^ This initiative should be integrated with the local operations of the vital statistics system, to generate routine, low-cost, local measures of mortality.

The observation of more registered deaths among men than among women needs to be further investigated. It remains unclear if this represents a relatively low probability of registration for women and/or true sex differences in the levels of mortality. There are limitations in our estimates of registration completeness, because the indirect demographic technique^26^ we used to estimate the completeness is based on several assumptions – e.g. constant fertility and mortality and zero net migration – that do not hold at state level in India. Some of the life expectancies estimated from the data in the vital statistics system are implausibly high – probably because of incomplete death registration. The life expectancies estimated from the sample registration system should be considered more reliable, given that system’s rigorous internal processes for verification and follow-up of death recording in each primary sampling unit.^40^ Incomplete registration was identified as a problem in previous assessments of civil registration in India.^41^ While the vital statistics system’s reporting coverage is high, greater attention is required to ensure the quality of the coverage in each registration unit.

The reporting of causes of death appears to be a major weakness of the vital statistics system. There is scope to increase the coverage of the certification scheme in several states, as well as to improve the reporting compliance of both government and private health facilities. Also, classification of all deaths by place of usual residence should improve the derivation of mortality indicators at state, district and even community levels. Greater emphasis is required for improving the quality of the medical certification of causes of death and reducing the large numbers of deaths that – despite the availability of a detailed manual on cause of death certification^34^ – are assigned ill-defined causes. Poor cause of death certification has even been observed in India’s teaching hospitals.^41^^,^^42^ Comparisons between the causes of death reported on the certification scheme’s forms and those derived via an expert physician review of medical records are needed. The current design and operational status of the vital statistics system provide a suitable platform for launching a programme to improve the data available for mortality measurement even at district level. Given its central role in the notification of vital events, determination of causes of death and compilation and use of the registration of data,^43^ India’s health sector could and should have a key role in strengthening the national vital statistics system. We need further analyses of the system’s performance to guide the system’s strengthening.

Previous research has indicated that, for a population with India’s demographic characteristics and mortality patterns, detailed information on approximately 20 000 deaths per state to measure cause-specific mortality reliably by age and sex, is needed.^44^ Therefore, detailed information on about 0.7 million deaths for India as a whole is needed. Such a sample is potentially available within the existing sampling frame of the vital statistics system’s registration units. In rural areas, a representative sample of primary health centres could be selected in each state. All home and in-facility deaths registered from the catchment areas of the selected health centres could then be followed up to ascertain causes of death – via verbal autopsies by health centre staff and by examination of the forms of the certification scheme, respectively. For urban areas, the sample could comprise a selection of municipal wards. In the future, computerization of the vital statistics system and the records of the certification scheme should enhance data compilation and analysis, especially when combined with the accurate recording of place of usual residence. If we are to have reliable mortality statistics for India, state-level plans need to be supported by greater intersectoral coordination, improvements in the training of human resources and the general strengthening of infrastructure.

##

**Box 2. Recommendations to strengthen the components of the civil registration and vital statistics system, India, 2015**

**Legal framework**

- Mandate completion of death reports before cremation or burial and for maintenance of records at all cremation and burial grounds; emphasize that death reports must be completed before the cremation or burial of fetuses, infants and children.
- Ask Office of the Registrar General of India to instruct state chief registrars to monitor civil registration and vital statistics system compliance across all districts. (Recommendation proposed in the 2012 *Report of the Committee on Strengthening of Civil Registration System*.)^37^

**Structure and organization**

- In each state, ask Office of the Registrar General of India’s state-level director of census operations to depute one official to liaise and coordinate civil registration and vital statistics system activities with the state chief registrar. (Recommendation proposed in the 2012 *Report of the Committee on Strengthening of Civil Registration System*.)^37^
- Use the National Health Mission’s resources – e.g. personnel, information technology equipment, printing services and public awareness campaigns – to support civil registration and vital statistics system operations in all states, whether or not the health sector is responsible for registration at any level.
- Establish interdepartmental coordination committees at state and district level to monitor intersectoral collaboration, evaluate performance and implement strengthening mechanisms for the civil registration and vital statistics system.

**System design**

- Design protocols for the reporting, registration and ascertainment of cause of death of individuals found dead on arrival at hospitals.
- Provide resources and protocols for household enquiries into cause of death in follow-up to death reporting and registration; such enquiries should use standardized formats and be conducted by designated government health staff.
- Develop standardized requirements for coding and classification of causes of death in the medical certification of cause of death scheme.
- Produce and supply standardized civil registration and vital statistics system software at district level for data entry, data archiving and processing for all stillbirths, live births and deaths. (Recommendation proposed in the 2012 *Report of the Committee on Strengthening of Civil Registration System*.)^37^

**Data management and quality control**

- Develop revised standards for statistical compilation in terms of age group, place of occurrence of event, place of usual residence and, where applicable, multiple causes per death.
- Develop standard framework to evaluate data quality at district level – including reporting coverage, timeliness and data accuracy.
- Promote the triangulation of district-level civil registration and vital statistics system data on vital events with related data from other sources – e.g. police or health programme records.
- Encourage feedback on local vital statistics to registration units and state-level registrar, to strengthen and monitor data quality.
- Strengthen the civil registration and vital statistics system supervisory role of sub-district and district-level registrars and designated health sector officials in states where the health department is not directly responsible for civil registration and vital statistics system operations. (Recommendation proposed in the 2012 *Report of the Committee on Strengthening of Civil Registration System*.)^37^
- Design and implement operations research activities at state level, for the empirical evaluation of the completeness of death registration and the validity of causes of death.
- Initiate collection and compilation of vital statistics in adequate samples for robust mortality measurement at state level on annual basis.

**Human resources**

- Make qualified personnel and information technology infrastructure available at all levels – but especially in registration units and the offices of district registrars. (Recommendation proposed in the 2012 *Report of the Committee on Strengthening of Civil Registration System*.)^37^
- At all levels, promote training to support reforms in structure, system design and data management processes.
- Train field staff in household cause-of-death enquiries, medical certification and coding of causes of death using International Classification of Diseases.

**Political will and support**

- At national level, engage with the Unique Identification Authority of India to enhance civil registration and vital statistics system performance.
- Conduct workshops for health bureaucrats and planning department staff on civil registration and vital statistics system data quality and vital statistics, and gain political support and advocacy for directing resources for civil registration and vital statistics system reforms and strengthening initiatives.
- Invite participation and collaboration from development partners and other stakeholders with interest in the civil registration and vital statistics system. (Recommendation proposed in the 2012 *Report of the Committee on Strengthening of Civil Registration System*.)^37^

**Public awareness and participation**

- Organize special registration events – with suitable local publicity – to facilitate completion of delayed registration.
- Promote local networks of notifiers for death registration – particularly for stillbirths, deaths among infants, women and the elderly – who could liaise with bereaved relatives and assist in their compliance with registration requirements.
- Mobilize participation in birth registration as an official requirement for school enrolment. (Recommendation proposed in the 2012 *Report of the Committee on Strengthening of Civil Registration System*.)^37^
